# Supplementary material for: A bite force database of 654 insect species
Source: Sci Data. 2024 Jan 10;11:58. doi: 10.1038/s41597-023-02731-w (PMC10781734; doi:10.1038/s41597-023-02731-w)
Supplement: Supplementary file 1 — Supplementary Information [file 41597_2023_2731_MOESM1_ESM.docx]

A bite force database of 654 insect species

Peter T. Rühr, Carina Edel, Melina Frenzel, Alexander Blanke

**SUPPORTING INFORMATION**


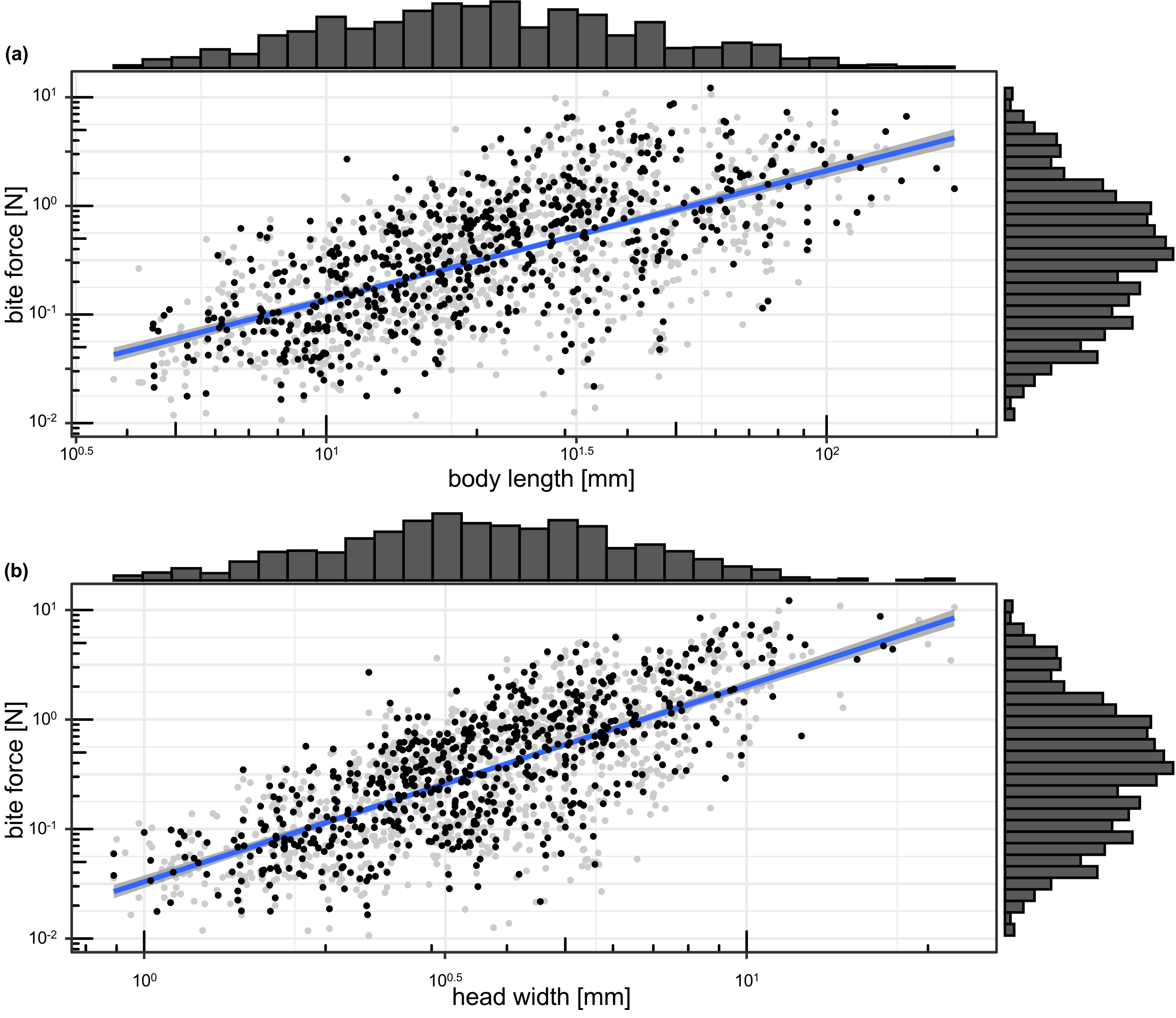


**Supplementary Figure 1:** Maximum bite force against body length (a) and head width (b). Grey dots show means of all maximum bite forces per specimen, black dots show means of all length measurements and maximum bite forces of all specimens per species. Marginal histograms at the x- and y-axes show mean size and mean bite force distribution per specimen, respectively. Regression lines and coefficients refer to log10-linear models of species-wise bite force against body length (a) or head width (b). All axes are log10-transformed.
